# Neuroinflammation and protein aggregation co-localize across the frontotemporal dementia spectrum

**DOI:** 10.1093/brain/awaa033

**Published:** 2020-03-17

**Authors:** W Richard Bevan-Jones, Thomas E Cope, P Simon Jones, Sanne S Kaalund, Luca Passamonti, Kieren Allinson, Oliver Green, Young T Hong, Tim D Fryer, Robert Arnold, Jonathan P Coles, Franklin I Aigbirhio, Andrew J Larner, Karalyn Patterson, John T O’Brien, James B Rowe

**Affiliations:** a1 Department of Psychiatry, University of Cambridge, Cambridge, UK; a2 Cambridge University Department of Clinical Neurosciences and Cambridge University Hospitals NHS Foundation Trust, Cambridge, UK; a3 Medical Research Council Cognition and Brain Sciences Unit, University of Cambridge, Cambridge, UK; a4 Istituto di Bioimmagini e Fisiologia Molecolare (IBFM), Consiglio Nazionale delle Ricerche (CNR), via Fratelli Cervi, Milano, Italy; a5 Department of Pathology, Cambridge University Hospitals NHS Foundation Trust, Cambridge, Cambridge, UK; a6 Wolfson Brain Imaging Centre, University of Cambridge, Cambridge, UK; a7 Division of Anaesthesia, University of Cambridge, UK; a8 Department of Neurology, The Walton Centre, Liverpool, UK

**Keywords:** microglia, tau imaging, semantic dementia, primary progressive aphasia, neuropathology

## Abstract

The clinical syndromes of frontotemporal dementia are clinically and neuropathologically heterogeneous, but processes such as neuroinflammation may be common across the disease spectrum. We investigated how neuroinflammation relates to the localization of tau and TDP-43 pathology, and to the heterogeneity of clinical disease. We used PET *in vivo* with (i) ^11^C-PK-11195, a marker of activated microglia and a proxy index of neuroinflammation; and (ii) ^18^F-AV-1451, a radioligand with increased binding to pathologically affected regions in tauopathies and TDP-43-related disease, and which is used as a surrogate marker of non-amyloid-β protein aggregation. We assessed 31 patients with frontotemporal dementia (10 with behavioural variant, 11 with the semantic variant and 10 with the non-fluent variant), 28 of whom underwent both ^18^F-AV-1451 and ^11^C-PK-11195 PET, and matched control subjects (14 for ^18^F-AV-1451 and 15 for ^11^C-PK-11195). We used a univariate region of interest analysis, a paired correlation analysis of the regional relationship between binding distributions of the two ligands, a principal component analysis of the spatial distributions of binding, and a multivariate analysis of the distribution of binding that explicitly controls for individual differences in ligand affinity for TDP-43 and different tau isoforms. We found significant group-wise differences in ^11^C-PK-11195 binding between each patient group and controls in frontotemporal regions, in both a regions-of-interest analysis and in the comparison of principal spatial components of binding. ^18^F-AV-1451 binding was increased in semantic variant primary progressive aphasia compared to controls in the temporal regions, and both semantic variant primary progressive aphasia and behavioural variant frontotemporal dementia differed from controls in the expression of principal spatial components of binding, across temporal and frontotemporal cortex, respectively. There was a strong positive correlation between ^11^C-PK-11195 and ^18^F-AV-1451 uptake in all disease groups, across widespread cortical regions. We confirmed this association with post-mortem quantification in 12 brains, demonstrating strong associations between the regional densities of microglia and neuropathology in FTLD-TDP (A), FTLD-TDP (C), and FTLD-Pick's. This was driven by amoeboid (activated) microglia, with no change in the density of ramified (sessile) microglia. The multivariate distribution of ^11^C-PK-11195 binding related better to clinical heterogeneity than did ^18^F-AV-1451: distinct spatial modes of neuroinflammation were associated with different frontotemporal dementia syndromes and supported accurate classification of participants. These *in vivo* findings indicate a close association between neuroinflammation and protein aggregation in frontotemporal dementia. The inflammatory component may be important in shaping the clinical and neuropathological patterns of the diverse clinical syndromes of frontotemporal dementia.

## Teaser

Bevan-Jones, Cope *et al.* report that neuroinflammation co-localizes with protein aggregation in all major types of frontotemporal dementia, both *in vivo* with positron emission tomography, and at post mortem. *In vivo* neuroinflammation patterns are disease-specific and can accurately classify patients into groups.

## Introduction

Frontotemporal dementia (FTD) encompasses a clinically and pathologically heterogeneous group of neurodegenerative conditions, including the behavioural variant (bvFTD) (Rascovsky *et al.*, 2011), non-fluent variant primary progressive aphasia (nfvPPA) and semantic variant primary progressive aphasia (svPPA) (Gorno-Tempini *et al.*, 2011). In recent years, attention has focused on understanding the pathogenic role of protein misfolding and aggregation, which is a cardinal feature of the post-mortem diagnostic criteria for frontotemporal lobar degeneration (FTLD) (Mackenzie *et al.*, 2010). However, there are several different pathological proteins and aggregation morphologies in FTD, with generally weak correlations between clinical syndrome and the type of pathological protein (Seelaar *et al.*, 2011) [with the exception of svPPA, which is strongly associated with TAR DNA-binding protein 43 (TDP-43) type C neuropathology] (Spinelli *et al.*, 2017). However, other neuropathological processes may be present in common across these diverse clinical syndromes and present potential therapeutic targets. In particular, there is converging evidence for the role of neuroinflammation in neurodegenerative dementias, including FTD, from genetic associations (Guerreiro *et al.*, 2013; Rayaprolu *et al.*, 2013; Broce *et al.*, 2018), CSF (Sjogren *et al.*, 2004; Woollacott *et al.*, 2018), epidemiology (Miller *et al.*, 2013, 2016), post-mortem tissue (Venneti *et al.*, 2008; Lant *et al.*, 2014) and animal models (Yoshiyama *et al.*, 2007; Bhaskar *et al.*, 2010; Yin *et al.*, 2010). Both the intensity of neuroinflammation and its distribution across the brain may be relevant determinants of the clinical syndrome. Here we aim to build on recent *in vivo* studies of Alzheimer’s disease, which demonstrate that neuroinflammation correlates spatially with tau aggregation (Dani *et al.*, 2018), by assessing whether this association extends to FTD, which is associated with many different conformations of pathological tau, or other protein aggregates such as TDP-43.

PET allows the topographic quantification of specific molecules using radioligands. In this study, we measured neuroinflammation and protein aggregation *in vivo* in patients with bvFTD, svPPA and nfvPPA, to answer key questions regarding the relationship of these pathophysiological processes. ^11^C-PK-11195, which binds to the translocator protein (TSPO) that is expressed on the outer mitochondrial membrane of activated microglia, is a robust and sensitive marker of microglial activation with an established role as a proxy for neuroinflammation in neurodegenerative diseases (Stefaniak and O’Brien, 2016). ^18^F-AV-1451 was originally developed to bind to paired helical filament tau in Alzheimer’s disease (Zhang *et al.*, 2012; Chien *et al.*, 2013; Xia *et al.*, 2013), and has been extensively used in Alzheimer’s and non-Alzheimer’s diseases. Elevated *in vivo* binding is seen in tauopathies characterized by straight filaments (Bevan-Jones *et al.*, 2016; Passamonti *et al.*, 2018; Smith *et al.*, 2017; Jones *et al.*, 2018), albeit with generally lower binding affinity than in Alzheimer’s disease, and also in TDP-43-related disease (Bevan-Jones *et al.*, 2018*a*, 2018*b*; Makaretz *et al.*, 2018). It also has low affinity for amyloid-β and α-synuclein (Xia *et al.*, 2013). Therefore, although the molecular interpretation of increased binding is incompletely understood (Marquié *et al.*, 2015; Sander *et al.*, 2016), this elevated *in vivo* binding suggests that ^18^F-AV-1451 represents a proxy index of aggregated non-amyloid-β pathological proteins across the FTD spectrum.

Given the evidence for differences in affinity of ^18^F-AV-1451 for different tau and TDP-43 conformational targets, our analysis strategy concentrates on the relative topographical distribution of binding across regions within each individual, rather than the simple magnitude of binding. In this way, we explicitly control for difference in binding affinity between syndromes and protein strains within each syndrome.

We test the hypotheses that, in FTD, neuroinflammation and protein aggregation are both increased in frontotemporal regions compared to controls, and that neuroinflammation and protein aggregation regionally co-localize in each FTD syndrome, consistent with the syndrome-specific neuropathological distributions (e.g. co-localization of neuroinflammation and protein aggregation in the temporal pole of patients with svPPA). We use data-driven approaches to PET imaging to elucidate the spatial modes of neuroinflammation associated with FTD, and machine learning based on multi-dimensional scaling of distributional dissimilarities, to investigate whether the cortical distribution of neuroinflammation and protein aggregation can accurately discriminate diagnostic groups thereby illustrating their mechanistic importance. The association between protein aggregation (tau or TDP-43) and inflammation (microglia) in the imaging data is supported by immunohistochemistry of post-mortem tissue from patients with FTD associated with FTLD-TDP types A and C, and FTLD-Pick’s disease.

## Materials and methods

As part of the NIMROD study (Bevan-Jones *et al.*, 2017), 31 patients (10 with bvFTD, 11 with svPPA and 10 with nfvPPA) underwent PET scanning with ^18^F-AV1451. Twenty-eight of the 31 (nine with bvFTD, nine with svPPA and 10 with nfvPPA) also underwent a PET scan with ^11^C-PK-11195. The order of scans was randomized. Fourteen healthy control participants underwent ^18^F-AV-1451 PET and, to minimize radiation exposure in healthy individuals, a different group of 15 healthy participants underwent ^11^C-PK-11195 PET scanning. Genetic and amyloid status (by PET or CSF biomarkers) for patients were tested if clinically indicated.

PET with ^18^F-AV-1451 and ^11^C-PK-11195 was performed on a GE Discovery 690 PET/CT (GE Healthcare) with a low dose CT for attenuation correction or on a GE Advance PET scanner (GE Healthcare) with a 15-min 68Ge/68Ga transmission scan for attenuation correction. The PET scan itself used dynamic imaging for 90 (^18^F-AV-1451) and 75 (^11^C-PK-11195) min, respectively. All radioligands were prepared at the Wolfson Brain Imaging Centre (WBIC), University of Cambridge, with high radiochemical purity (>95%). Each subject underwent contemporaneous 3T MRI using a Siemens Magnetom Skyra, Verio or Tim Trio (www.medical.siemens.com). A high-resolution T_1_-weighted sequence was acquired (176 slices of 1.0 mm thickness, echo time = 2.98 ms, repetition time = 2300 ms, flip angle = 9°, acquisition matrix 256 × 240; voxel size = 1 × 1 × 1 mm^3^) and used for tissue segmentation (grey and white matter along with CSF), and for non-rigid registration of standard space regions of interest. For both ligands, non-displaceable binding potential (BP_ND_) was calculated in 83 regions of interest, defined by a Hammers atlas modified to include the midbrain and the dentate nucleus of the cerebellum, by kinetic modelling using a simplified reference tissue model, with cerebellar grey matter as reference region for ^18^F-AV-1451 (Passamonti *et al.*, 2018) and supervised cluster analysis used to define the ^11^C-PK-11195 reference region (Yaqub *et al.*, 2012). Prior to kinetic modelling, all region of interest data were corrected for CSF contamination of the region (i.e. partial volume corrected) through division by the mean region grey plus white matter fraction, determined using tissue probability maps smoothed to PET spatial resolution.

Four data analysis approaches were used, each designed to answer a different focused question and to explicitly control for expected between-subject and between-region differences in ligand affinity.

As a first-stage data exploration of between-group differences, a repeated-measures ANOVA was performed across the 83 regions, including age as a covariate and Greenhouse-Geisser penalization of degrees of freedom to correct for non-sphericity. *Post hoc t*-tests were then performed between each group, corrected for false discovery rate (FDR) over regions.

Second, to examine the relationship between neuroinflammation and protein aggregation in each disease group, a correlation between the regional BP_ND_ of each ligand was performed. PET scanning with any ligand characteristically results in a general pattern of lower BP_ND_ in brain regions such as temporal lobe and higher BP_ND_ in deep brain nuclei. We were concerned that such non-specific effects might drive apparent correlations, and weak correlations were observed between our cohorts of controls for each ligand (Supplementary Fig. 1). To control for this, we examined the between-ligand correlation within each disease group both with and without subtraction of the control mean BP_ND_ for each of the 83 regions of interest. We then assessed whether the association between the regional BP_ND_ of the two ligands was independent of atrophy. To do this, we first calculated a combined grey and white matter volume *t*-score for each brain region and disease group, representing the degree of atrophy in that region compared to controls (we observed that this combined grey and white matter *t*-score was more strongly related to ligand binding than either tissue class alone, and therefore report this as the most conservative correction and informative association). We then calculated partial correlations between the two ligands, with the effect of atrophy partialled out, as well as between each ligand and atrophy individually, with the effect of the other ligand partialled out.

Third, to elucidate the topographical patterns of inflammation and protein aggregation in FTD, we entered the BP_ND_ of each ligand in each of the 83 regions of interest into a principal component analysis. Components were retained by Cattell’s criterion (i.e. to the elbow of the scree plot) and then tested for group differences across diagnosis in a repeated measures ANOVA. *Post hoc t*-tests examined group differences in the expression of each topographical pattern. These first three analyses were performed in SPSS Statistics version 25 (IBM).

Finally, we undertook an analysis of the relative distribution of ligand binding potential for each ligand for every individual. This used previously published non-parametric methods (Bevan-Jones *et al.*, 2016), that were explicitly designed to control for between-subject differences in the scaling of each ligand, such as might result from differences in the affinity of ^18^F-AV-1451 for different conformations of tau or TDP-43, as well as spatial dependence between adjacent regions in PET data due to signal spread. These methods can be conceptualized as analogous to multi-voxel pattern analysis techniques for functional MRI (Kriegeskorte *et al.*, 2008), but rather than attempting to classify observed stimuli within an individual on the basis of their representational similarity, here we are attempting to classify individuals on the basis of the similarity of relative ligand BP_ND_ distributions within their brains, blinded to overall differences in binding affinity. To do this, for each ligand and every individual separately, the parcellated data were converted to 83-element linear vectors. For each ligand separately, the resultant vectors were non-parametrically correlated (Spearman’s rho) pairwise between individuals, resulting in two matrices that represented the similarity of each individual’s scan to each other individual for that ligand. The inverse of these matrices (i.e. the between-individual dissimilarities) were used to calculate a 2D scaling for each disease subgroup pair, using the squared metric stress distance criterion of the ‘mdscale’ function in MATLAB R2017b (Mathworks). The resulting locations in 2D space formed the inputs to a 10-fold cross-validated linear support vector machine (CV-SVM) for between-group classification based on each ligand separately. Statistical significance of the classification was assessed by comparison of the loss function of the CV-SVM against a null distribution of loss functions created by 1000 repetitions of the same procedure for identical data but shuffled group assignment labels. For those individuals who underwent scanning with both ligands, the CV-SVM process was repeated on multi-modal, 4D scaling. To confirm that our machine learning results were not significantly influenced by age or sex, we attempted supplementary classifications based on these factors.

Additionally, we performed quantitative immunohistochemistry on 12 post-mortem cases from the Cambridge Brain Bank to augment the imaging results. From our database of specimens, we selected three cases of FTLD-Pick's (a tauopathy), three cases of FTLD-TDP type C and three cases of FTLD-TDP type A, and compared these to three cases of Braak stage V Alzheimer’s disease. For each case, fixed brain tissue was sampled from the prefrontal cortex [Brodmann area (BA) 44], middle temporal cortex (BA22/21), parietal cortex (BA7), and occipital cortex (BA17/18). These were embedded in paraffin, and sectioned at 10 µm. For each region, two neighbouring sections were stained by immunohistochemistry using antibodies directed against CD68 (clone PG-M1, Dako), a marker of microglia and macrophages, and against the relevant pathological protein: hyperphosphorylated tau (AT8, MN1020, Thermo Scientific) or TDP43 (TIP-PTD-P02, Cosmo Bio Co Ltd.).

The number of protein aggregates, microglia, and cell nuclei were counted in series of fields of view placed uniformly randomly onto each section: a virtual grid with uniform distances between lines in the *x* and *y* direction, 1875 µm × 1875 µm, was superimposed onto each section. The position of fields of view was at the intersections of the grid lines, but only where lines crossed overlapping grey matter. The fields of view were 125 µm × 125 µm and counting was done under a 40× objective lens.

A microglial cell was counted when CD68 reactivity was visible over or around a cell nucleus. Based on the morphology, cells positive for the CD68 staining were divided into ramified microglia, amoeboid microglia and macrophages. In FTLD-Pick's, Pick bodies and glial tau inclusions were counted. In FTLD-TDP inclusions type A and C, dystrophic neurites, and lentiform intranuclear inclusions and neuronal cytoplasmic inclusions, respectively were counted. In Alzheimer’s disease, neurofibrillary tangles and neuritic plaques were counted. The densities of pathology, microglia and cell nuclei were calculated by dividing the total counts by the area in which they were counted and expressed per square millimetre. Analyses of the relationship between protein pathology and microglia were controlled for atrophy by partialling out the density of cell nuclei in correlations, and accounting for this as a co-variate of no interest in generalized linear models.

### Data availability

The data that support the findings of this study are available from the corresponding author, upon reasonable request.

## Results

Summary demographics are outlined in Table 1, and neuropsychological test scores, motor features, genetic and CSF status for each participant are provided in Table 2. Within the bvFTD group, two patients were positive for pathogenic mutations in the microtubule associated protein tau (MAPT) and three for expansions in C9 open reading frame 72 (C9ORF72). One of the nfvPPA group had a mutation in progranulin (GRN). CSF or PET amyloid status was assessed in six participants (four with svPPA, and two with nfvPPA), all of whom were negative.

**Table 1:** Summary demographics and neuropsychometry

Group	*n*	**M:F** *	**Mean age** ^#,$^	Education, years*^,#,$^	ACE-R /100*^,#,$,**†**^	**MMSE/30** ^,#,$^	FAB/18	FTDRS_Logit	FTDRS_Percent/100
nfvPPA	10	3:7	71	12	79	27	11	1.92	71.7
svPPA	11	9:2	68	14	63	25	12	0.74	52.8
bvFTD	10	5:5	60	13	57	22	8	−2.46	17.4
Tau controls	14	7:7	67	16	95	29	–	–	–
PK controls	15	7:8	69	14	92	29	–	–	–

Pairwise comparisons are by *t*-test for each demographic except sex comparison by χ^2^.

* *P < *0.05 significant pairwise comparison nfvPPA versus combined control group.

# *F*-test significant *P < *0.05 across all groups.

$ *P < *0.05 significant pairwise comparison bvFTD versus combined control group.

† *P < *0.05 significant pairwise comparison svPPA versus combined control group,

F = female; FAB = Frontal Assessment Battery; FTDRS = FTD Rating Scale; M = male; MMSE = Mini-Mental Status Examination.

**Table 2:** Demographics, neuropsychological testing, genetic/amyloid status and motor phenotype for each disease participant

Case	Diagnosis	Gene/amyloid status	Sex	Entry age	Education, years	ACE-R /100	MMSE/30	FAB/18	FTDRS Logit score	Motor features
1	nfvPPA	Aβ−ve (CSF)	M	55	14	93	29	15	3.35	−
2	nfvPPA	–	F	67	16	88	28	13	2.19	−
3	nfvPPA	Aβ−ve (CSF)	F	62	11	90	27	15	1.92	+
4	nfvPPA	–	F	84	11	85	30	11	5.39	+
5	nfvPPA	–	F	81	10	78	28	15	0.16	−
6	nfvPPA	–	F	74	10	40	16	7	−0.8	−
7	nfvPPA	*GRN*	M	66	10	76	22	9	−0.2	−
8	nfvPPA	–	F	77	11	86	30	13	0.34	+
9	nfvPPA	–	M	74	11	87	30	10	1.47	+
10	nfvPPA	–	F	70	11	71	25	6	5.39	−
11	svPPA	–	M	77	16	45	22	11	−1.27	−
12	svPPA	–	M	69	16	77	28	11	−1.54	−
13	svPPA	Aβ−ve (CSF)	M	61	15	79	30	16	−0.4	−
14	svPPA	Aβ−ve (PiB)	F	65	18	72	27	16	–	−
15	svPPA	Aβ−ve (CSF)	M	67	17	71	27	17	2.49	−
16	svPPA	Aβ−ve (CSF)	M	65	13	68	27	13	5.39	−
17	svPPA	–	M	72	13	63	25	12	1.26	−
18	svPPA	–	F	63	10	59	26	14	0.7	−
19	svPPA	–	M	69	18	85	30	14	2.19	−
20	svPPA	–	M	63	10	61	27	8	−0.8	−
21	svPPA	–	M	72	9	9	3	0	−0.59	−
22	bvFTD	–	F	63	12	79	29	11	−3.09	−
23	bvFTD	–	M	61	11	47	15	5	−2.18	−
24	bvFTD	*MAPT*	F	50	16	43	21	9	−3.8	−
25	bvFTD	–	M	75	16	68	21	6	−0.4	+
26	bvFTD	*MAPT*	F	70	16	38	14	7	−3.09	−
27	bvFTD	–	F	67	11	71	28	8	−0.8	−
28	bvFTD	–	M	51	14	81	29	11	−2.58	−
29	bvFTD	*C9orf72*	M	56	10	53	25	6	−1.03	+
30	bvFTD	*C9orf72*	F	51	10	41	16	7	−3.8	−
31	bvFTD	*C9orf72*	M	58	9	46	17	5	−3.8	−

Aβ−ve = negative tests for amyloid-β by CSF biomarkers or PiB PET scan. ACE-R = Addenbrooke’s Cognitive Examination Revised; FAB = Frontal Assessment Battery; FTDRS = FTD Rating Scale; MMSE = Mini-Mental Status Examination; PiB = Pittsburgh compound B.

### Group comparisons of frontotemporal dementia with controls

**Figure 1:**
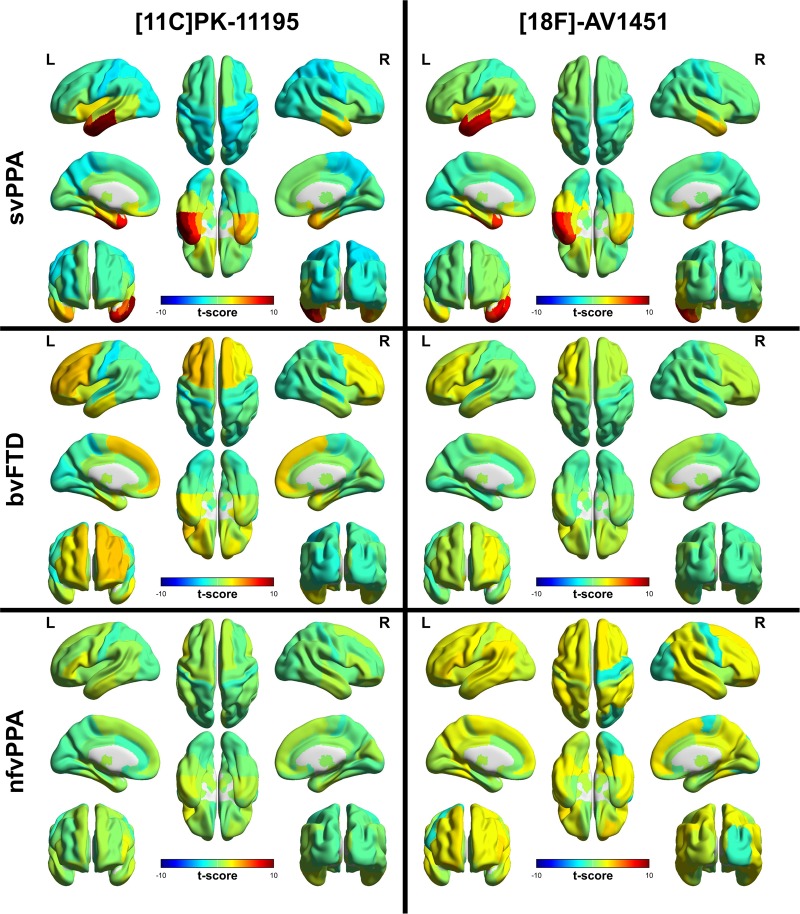
**Regional ligand binding by group.** Unthresholded regional *t*-scores for each disease group compared to the control group for ^11^C-PK-11195 BP_ND_ on the *left* and ^18^F-AV-1451 BP_ND_ on the *right*.

The repeated-measures ANOVA of regional ^11^C-PK-11195 binding across the FTD groups and controls, controlled for age and sex, demonstrated a significant interaction between region and diagnosis [*F*(37.4,423.5) = 3.54, *P *<* *0.001]. T-maps from the *post hoc* pairwise comparisons between the control group and each disease group are shown in Fig. 1. After correction for FDR, regions with significantly elevated binding were (i) in the bvFTD group: bilateral superior frontal gyri and putamen, right nucleus accumbens, left posterior orbital gyrus, inferior frontal gyrus and middle frontal gyrus; and (ii) in the svPPA group: left insula, middle and inferior temporal gyri, right superior parietal gyrus, middle and inferior temporal gyri, bilateral postcentral gyri, superior temporal gyrus, parahippocampal and ambient gyri, amygdala, inferior lateral anterior temporal lobe, medulla, nuclei accumbens, medial anterior temporal lobe, fusiform gyri. Left medial anterior and inferior lateral anterior temporal lobe, and superior, middle and inferior temporal gyri also survived Bonferroni correction. In the nfvPPA group no differences survived FDR correction but the peak *t*-score was in left inferior frontal gyrus [*t*(23) = 2.17, uncorrected *P *=* *0.04], which would be expected *a priori* to be the disease epicentre (Rogalski *et al.*, 2011).

The repeated measures ANOVA of regional ^18^F-AV-1451 binding across the FTD groups and controls, controlled for age and sex, showed a significant interaction between region and diagnosis [*F*(33.6,403.6) = 3.80, *P *<* *0.001]. T-maps from the *post hoc* pairwise comparisons between the control group and each disease group are shown in Fig. 1. After correction for FDR, significantly elevated binding was seen in svPPA, in the following regions: left amygdala, fusiform, medial anterior temporal lobe, middle and inferior temporal gyri and superior temporal gyrus, bilateral inferolateral anterior temporal lobes. Left medial anterior temporal lobe and middle and inferior temporal gyri also survived Bonferroni correction. For bvFTD and nfvPPA, the group differences did not survive FDR correction.

There was a trend towards patients with bvFTD due to C9ORF72 having globally higher ^11^C-PK-11195 binding than those with no genetic diagnosis, while those with abnormalities of MAPT had intermediate binding [main effect of gene *F*(2,3) = 8.26, *P *=* *0.060; Tukey’s HSD *P *=* *0.049 for C9ORF72 (mean = 0.176, ±0.017) versus no genetic diagnosis (mean = 0.094, ±0.016), but non-significant for either group versus MAPT (mean = 0.123, ±0.018)]. There was no significant interaction between genetic diagnosis and region [*F*(5.3,7.8) = 1.47, *P *=* *0.300].

There was no effect of bvFTD genetic status on ^18^F-AV-1451 binding [main effect of gene *F*(2,4) = 0.85, *P *=* *0.492; interaction between gene and regional binding *F*(6.2,12.3) = 1.78, *P *=* *0.183].

For the patients with nfvPPA, the single patient with GRN mutation was not significantly different from those without a genetic diagnosis in binding of either ^11^C-PK-11195 [main effect of gene *F*(1,6) = 0.38, *P *=* *0.559; interaction between gene and regional binding *F*(4.8,28.7) = 1.17, *P *=* *0.349] or ^18^F-AV-1451 [main effect of gene *F*(1,6) = 0.1.66, *P *=* *0.244; interaction between gene and regional binding *F*(3.2,19.2) = 0.47, *P *=* *0.720].

### Correlation of ^11^C-PK-11195 with ^18^F-AV-1451 in frontotemporal dementia

**Figure 2:**
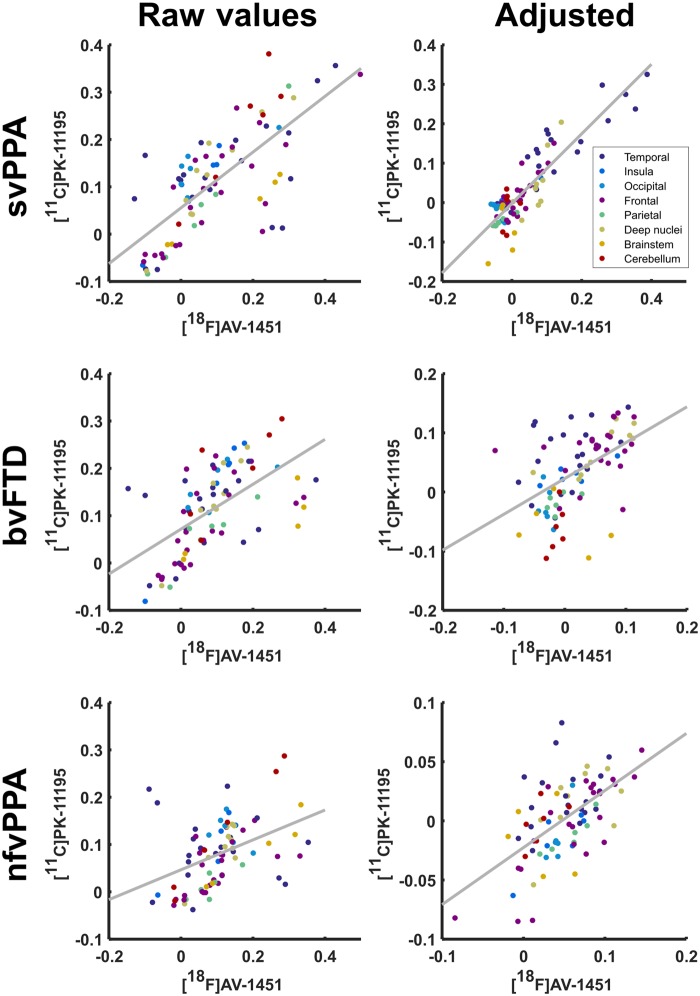
**Scatter plot of the regional mean BP_ND_ for ^11^C-PK-11195 against regional mean BP_ND_ of ^18^F-AV-1451 by disease group.** For each disease group raw values are demonstrated on the *left* with values adjusted for non-specific signal strength through subtraction of the regional control mean shown on the *right*.

Regional control-adjusted group mean ^11^C-PK-11195 BP_ND_ was strongly correlated with regional group mean ^18^F-AV1451 BP_ND_ in each group both before and after the subtraction of the control group values in every region: svPPA [*r*(81) = 0.727, *P *<* *0.0001 before, *r*(81) = 0.883, *P *<* *0.0001 after], bvFTD [*r*(81) = 0.582, *P *<* *0.0001 before, *r*(81) = 0.499, *P *<* *0.0001 after], and nfvPPA [*r*(81) = 0.427, *P *<* *0.0001 before, *r*(81) = 0.589, *P *<* *0.0001 after] (Fig. 2). These correlations were only slightly affected by partialling out the effect of regional brain atrophy. For svPPA *r*(80) = 0.634, *P *<* *0.0001 [partial correlation between AV and atrophy *r*(80) = −0.398, *P *=* *0.0002; PK and atrophy *r*(80) = −0.330, *P *=* *0.0024], for bvFTD *r*(80) = 0.459, *P *<* *0.0001 [partial correlation between AV and atrophy *r*(80) = −0.020, *P *=* *0.8576; PK and atrophy *r*(80) = −0.360, *P *=* *0.0009], and for nfvPPA *r*(80) = 0.542, *P *<* *0.0001 [partial correlation between AV and atrophy *r*(80) = −0.321, *P *=* *0.0033; PK and atrophy *r*(80) = −0.048, *P *=* *0.6722].

Finally, to ensure that our methods for controlling for non-specific general regional differences in PET tracer retention were adequate, we repeated our analysis in each lobe separately. Across all three patient groups combined (Supplementary Fig. 2), corrected for control binding, and accounting for group with ANCOVA, significant associations were observed between ^11^C-PK11195 and ^18^F-AV1451 BP_ND_ in temporal lobe *F*(1,54) = 82.11, *P *<* *0.0001 [diagnosis *F*(2,54) = 15.67, *P *<* *0.0001, interaction *F*(2,54) = 2.16, *P *=* *0.12], insula and cingulate *F*(1,12) = 29.18, *P *=* *0.0002 [diagnosis *F*(2,12) = 25.82, *P *<* *0.0001, interaction *F *<* *1], frontal lobe *F*(1,66) = 52.88, *P *<* *0.0001 [diagnosis *F*(2,66) = 32.31, *P *<* *0.0001, interaction *F*(2,66) = 7.41, *P *=* *0.0013], parietal lobe *F*(1,12) = 24.3, *P *=* *0.0003 [diagnosis *F*(2,12) = 10.45, *P *<* *0.0024, interaction *F *<* *1], and deep nuclei *F*(1,24) = 27.42, *P *<* *0.0001 [diagnosis *F*(2,24) = 8.07, *P *=* *0.0021, interaction *F*(2,24) = 3.4, *P *=* *0.05]. We did not find significant associations in occipital lobe *F *<* *1 [diagnosis *F *<* *1, interaction *F*(2,12) = 4.92, *P *=* *0.0275], cerebellum *F*(1,12) = 2.55, *P *=* *0.1366 [diagnosis *F*(2,12) = 1.98, *P *=* *0.1809, interaction *F *<* *1], or brainstem *F *<* *1 [diagnosis *F*(2,9) = 1.69, *P *=* *0.2388, interaction *F *<* *1].

### Principal component analysis of ^11^C-PK-11195 and ^18^F-AV-1451

**Figure 3:**
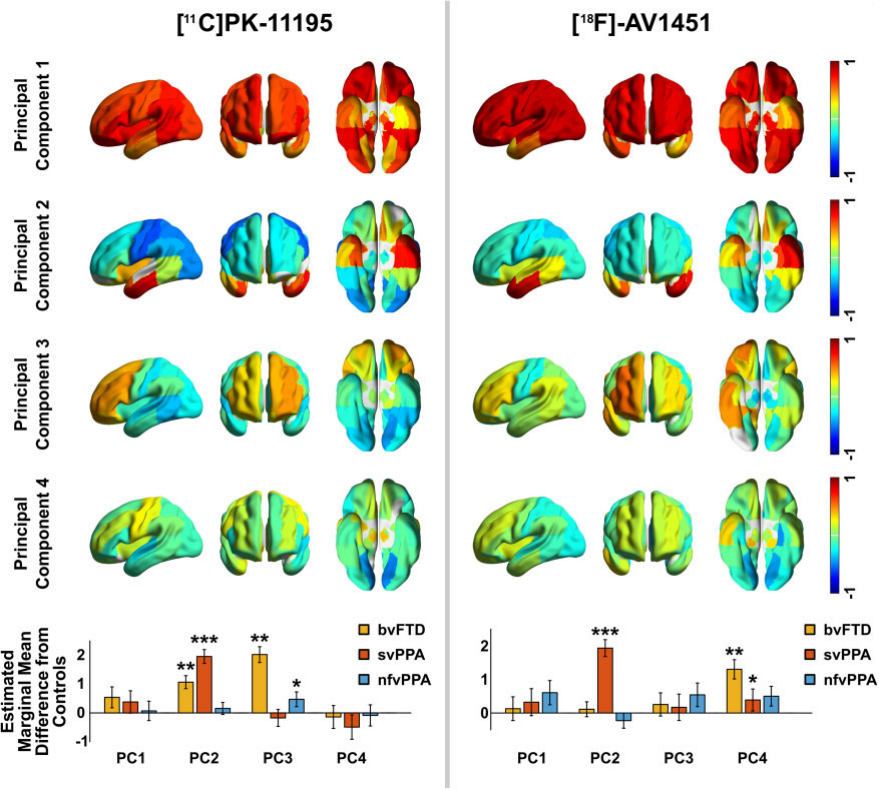
**First four principal components for ^11^C-PK-11195 and ^18^F-AV-1451.** ^18^F-AV-1451 component 5 was also retained by Cattell’s criterion but was not strongly weighted to any region and did not discriminate groups so is omitted here for parsimony. The *bottom* row shows, for each principal component (PC), the difference between each patient group and controls, adjusted for age and sex in the repeated measures ANOVA. Error bars span ± 1 SEM (standard error of the mean for the patient group). Significance in *post hoc* tests: ***P <* *0.001, **P <* *0.01, *P <* *0.05.

Four principal components were detected in the ^11^C-PK11195 BP_ND_ data before the elbow of the scree plot, which together explained 64% of the variance in the data (Fig. 3). Component 1 reflected whole brain binding. Component 2 was strongly weighted to the bilateral anterior temporal lobes. Component 3 primarily comprised frontal binding with a right-sided predominance. Component 4 was not strongly loaded onto any single region but was weighted towards motor cortex. In a repeated measures ANOVA including these four principal components, there was a main effect of diagnosis [*F*(3,39) = 11.07, *P *<* *0.001] and a significant interaction between principal component weighting and diagnosis [*F*(8.03, 104.36) = 4.16, *P *<* *0.001]. After correction for age and sex these remained significant [diagnosis *F*(3,34) = 8.98, *P *<* *0.001; interaction between principal component weighting and diagnosis *F*(8.14,92.28) = 4.11, *P *<* *0.001]; age and sex were not significant predictors [age *F*(1,34) = 0.40, *P *=* *0.533; sex *F*(1,34) = 1.45, *P *=* *0.237]. *Post hoc t*-tests between individual disease groups and controls showed svPPA was associated with an increase in component 2 [*t*(10.20) = 8.30, *P *<* *0.001], bvFTD associated with both increased component 2 [*t*(9.30) = 3.37, *P *=* *0.008] and component 3 [*t*(8.85) = 3.95, *P *=* *0.003] and nfvPPA associated with increased component 3 [*t*(12.4) = 2.38, *P *=* *0.034] (Fig. 3). Components 1 and 4 did not significantly differ between controls and patient groups.

Five principal components were detected in the ^18^F-AV-1451 BP_ND_ data before the elbow of the scree plot, which together accounted for 76% of the variance in the data. Component 1 again reflected global binding but less marked in the temporal poles, which were loaded onto component 2 (left) and component 4 (right). Component 3 was weighted asymmetrically towards frontal lobe binding. Component 5 was not strongly loaded onto any single region but was weighted towards bilateral superior temporal poles. In a repeated measures ANOVA including these five principal components, there was a main effect of diagnosis [*F*(3, 41) = 5.43, *P *=* *0.003] and a significant interaction between principal component weighting and diagnosis [*F*(11,150.6) = 3.68, *P *<* *0.001]. After correction for age and sex these remained significant [diagnosis *F*(3,36) = 3.80, *P *=* *0.018; interaction between principal component weighting and diagnosis *F*(10.9,130.5) = 3.33, *P *<* *0.001]; age was also a significant predictor [*F*(1,36) = 4.47, *P *=* *0.041] but sex was not [*F*(1, 36) = 0.11, *P *=* *0.743]. The bvFTD group had increased weightings in component 4 [*t*(11.58) = 3.28, *P *=* *0.007], and svPPA had increased weightings in component 2 [*t*(11.88) = 6.819, *P *<* *0.0001] and component 4 [*t*(12.9) = 2.48, *P *=* *0.028] (Fig. 3). There were no significant *post hoc* differences between nfvPPA and controls. Components 1, 3 and 5 did not differ significantly between controls and patient groups.

In summary (Fig. 3), patients with bvFTD demonstrated significantly elevated PK binding in spatial modes that included frontal lobe and temporal poles, and elevated AV binding in frontal regions; patients with svPPA demonstrated significantly elevated AV and PK binding in temporal poles; patients with nfvPPA demonstrated significantly elevated PK binding in frontal lobe, but to a lesser extent than patients with bvFTD, and they showed no spatial modes with elevated AV binding.

### Non-parametric analysis of ^11^C-PK-11195 and ^18^F-AV-1451 distributions

The principal component analyses suggest that a large amount of the variance between subjects relates to whole brain PET signal. While this might reflect global differences in protein aggregation and neuroinflammation, it could also be explained by variations in radioligand affinity for different protein pathologies or other non-specific influences discussed below. We therefore performed an analysis of the relative distribution of PET signal for each individual scan, blinded to differences in overall signal magnitude by non-parametric rank-order statistical methods.

**Figure 4:**
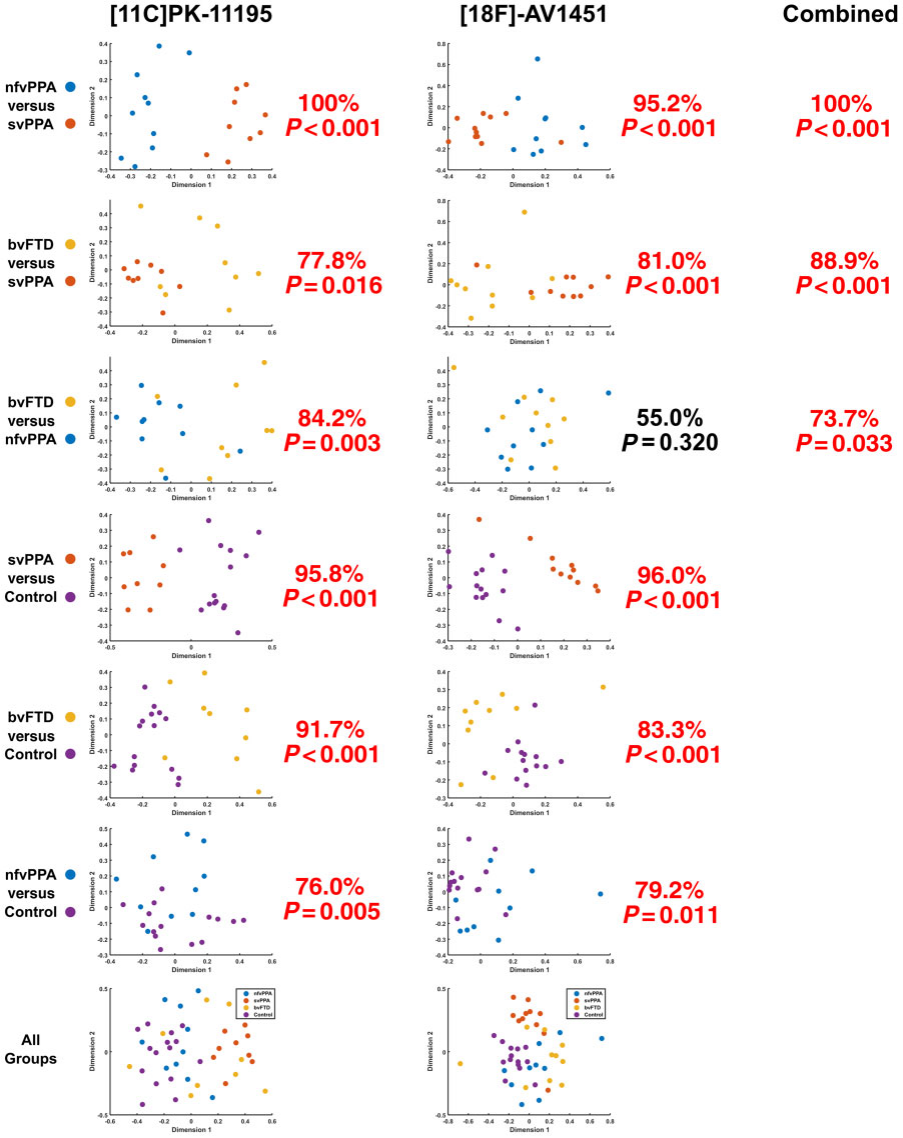
**Pairwise classification accuracy for each ligand.** ^11^C-PK-11195 (*left*), ^18^F-AV-1451 (*middle*), and using combined data (*right*). The graphs represent a 2D projection of the between-individual PET signal distribution dissimilarity calculated according to the squared metric stress criterion. A 10-fold cross-validated support vector machine was applied to each plot, and the classification accuracy compared to a null distribution of 1000 randomizations for non-parametric significant testing. For each comparison, percentage classification and *P*-value is stated. In simple terms, this means that the similarity of the distribution of ligand binding across the brain for each individual was assessed irrespective of the absolute magnitude of binding (and therefore not determined by differences in ligand affinity for different pathological subtypes). Note how in the *top left* plot (nfvPPA versus svPPA for ^11^C-PK-11195) two groups of patients are clearly separated. By contrast, in the second column third row (bvFTD versus nfvPPA for ^18^F-AV-1451) the points are intermingled, with only chance-level classification.

Multi-dimensional scaling plots of the non-parametric similarity between ligand distributions, for each subgroup pair and for all groups combined, are shown in Fig. 4. The CV-SVM classification accuracy and permutation-based statistical significance are indicated next to each plot. Classification was significantly better than chance in all cases, except for the finding that the non-parametric distribution of ^18^F-AV-1451 was unable to distinguish between bvFTD and nfvPPA.

For those FTD participants that underwent scanning with both ligands, the classification procedure was repeated after combining the multi-dimensional scaling data such that the CV-SVM operated on four dimensions rather than two. This resulted in an improvement in the differentiation of bvFTD and svPPA compared to either ligand alone (88.9% classification accuracy, *P *<* *0.001). Multimodal nfvPPA versus svPPA classification accuracy matched the performance of ^11^C-PK-11195 at 100%, *P *<* *0.001, but bvFTD versus nfvPPA classification performance was intermediate compared to each ligand alone, at 73.7%, *P *=* *0.033.

This method was not able to classify participants based on age or sex (Supplementary Fig. 3).

### Post-mortem immunohistochemistry

**Figure 5:**
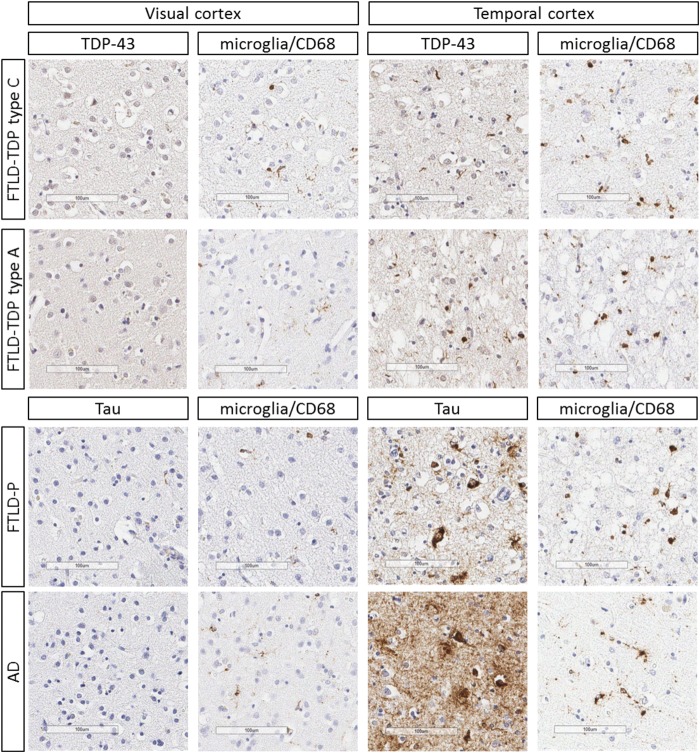
**Immunohistochemistry from cases with FTLD-TDP43 types A and C, FTLD-P (Pick’s disease), and Alzheimer’s disease (AD) at Braak stage V.** Areas of low (visual cortex BA17/18) and high (temporal cortex, BA21/22) disease burden are shown. Scale bars = 100 µm. Representative micrographs from the same location on adjacent sections stained for the relevant protein aggregate (phosphorylated-tau or TDP43) and CD68 (expressed by microglia), respectively.

**Figure 6:**
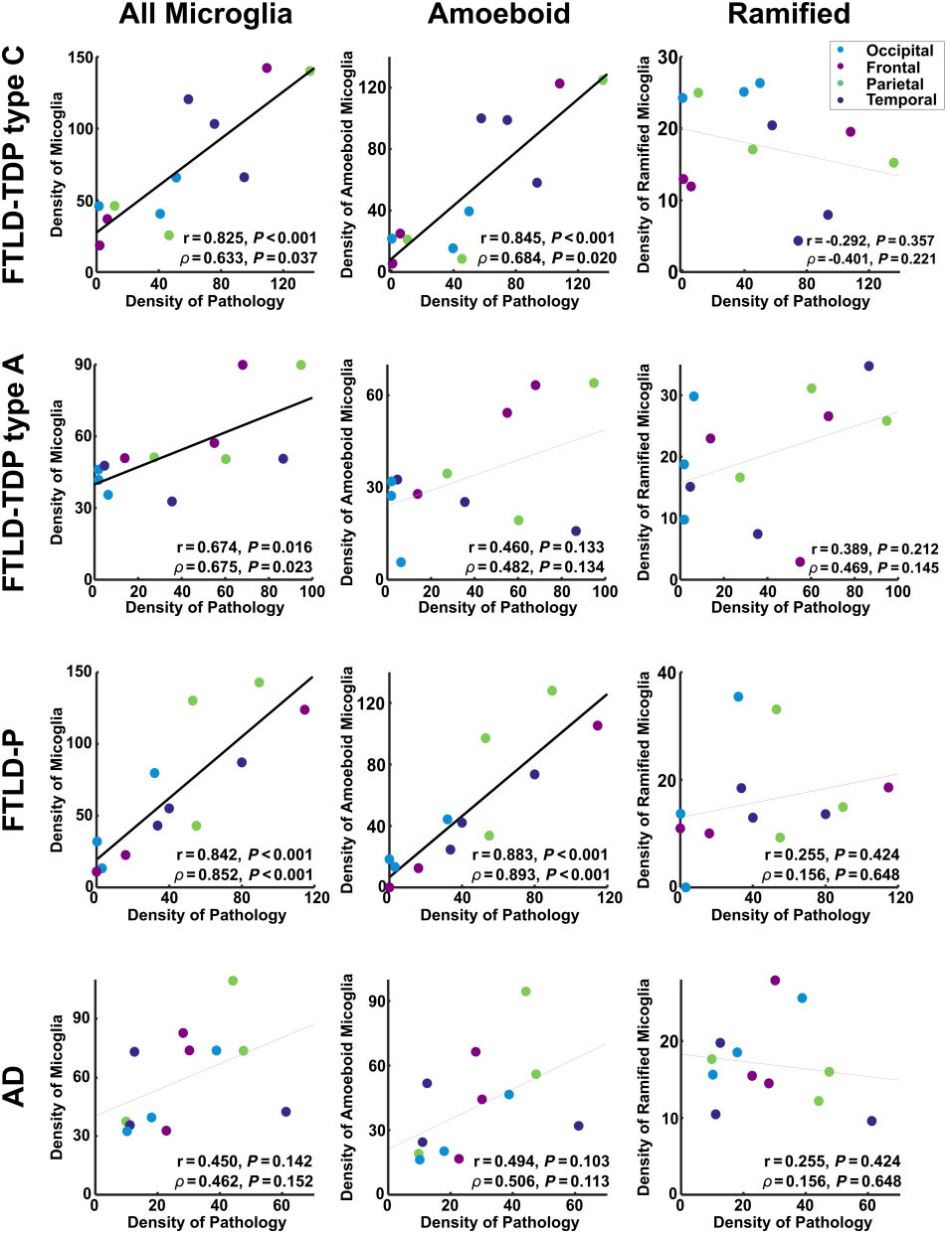
**The relationship between protein aggregation and microglia in each post-mortem sample.** Densities are quantified as the number of microglia or pathological inclusions per square millimetre. Each point represents a single brain region in a single individual. The Pearson correlation (r), and the partial correlation (*ρ*) after factoring out the density of cell nuclei are shown for each relationship, along with the corresponding *P*-value. Trend lines are emboldened when both correlation and partial correlation were significant at *α* < 0.05.

Representative post-mortem immunohistochemistry for protein aggregate inclusions and microglia are shown in Fig. 5. The quantitative relationship between protein aggregation and microglia is shown for each disease group individually in Fig. 6.

A generalized linear model demonstrated a significant relationship between the overall density of pathology and the density of microglia [Wald χ^2^(1) = 21.40, *P *<* *0.001], accounting for subject factors (including age and sex) nested within diagnostic group [Wald χ^2^(8) = 79.55, *P *<* *0.001], brain region [Wald χ^2^(3) = 18.06, *P *<* *0.001], diagnosis [Wald χ^2^(3) = 3.95, *P *=* *0.267], the interaction between diagnosis and brain region [Wald χ^2^(9) = 23.63, *P *=* *0.005], and the density of cell nuclei [Wald χ^2^(1) = 21.12, *P *<* *0.001].

Repeat generalized linear models with the same design demonstrated that this effect was driven by a significant relationship between the density of pathology and the density of amoeboid (rounded, phagocytic, activated) microglia [Wald χ^2^(1) = 30.83, *P *<* *0.001], but not the density of ramified (dendritic, resting) microglia [Wald χ^2^(1) = 0.02, *P *=* *0.898].

## Discussion

This *in vivo* study provides insights into complementary pathophysiological processes of FTD. Taken as a whole, our findings support an important role for neuroinflammation across the FTD spectrum, corroborating suggestions from epidemiological (Venneti *et al.*, 2008; Lant *et al.*, 2014), genetic (Guerreiro *et al.*, 2013; Rayaprolu *et al.*, 2013; Broce *et al.*, 2018), imaging (Cagnin *et al.*, 2004; Miyoshi *et al.*, 2010) and animal studies (Yoshiyama *et al.*, 2007; Bhaskar *et al.*, 2010; Yin *et al.*, 2010). Using regional ANOVA and a principal components analysis, we have shown that neuroinflammation (indexed by ^11^C-PK-11195) and protein aggregates (tau or TDP43, as indexed by ^18^F-AV-1451) are elevated in FTD (Figs 1 and 3). Furthermore, neuroinflammation is regionally co-localized with protein aggregation within the individual syndromes, including most strongly in svPPA, where the predominant aggregated protein inclusions are TDP-43 rather than tau (Fig. 2). Principal component analysis revealed distinct spatial modes of neuroinflammation, with frontotemporal, temporal pole and global distributions (Fig. 3). The weighting of these regional distributions differs between groups, supporting the regional differences in the pair-wise comparisons. The spatial modes of protein aggregation (Fig. 3) similarly reflect the well characterized distributions of pathology in each patient group. However, the distribution of protein aggregation appears to be less focal than neuroinflammation in nfvPPA. To identify distinctive information from the patterns of inflammation and aggregation, and control for the marked individual differences in ligand binding affinity in different subtypes of FTD, we used non-parametric multi-dimensional scaling and cross-validated linear support vector machines to classify patients. We demonstrated that the distribution of neuroinflammation can accurately distinguish the FTD syndromes from controls and from each other (Fig. 4). Classification was often possible based on the distribution of protein aggregation, but with less accuracy. The greater discriminatory ability of neuroinflammation emphasizes its potential mechanistic relevance to the pathophysiology of FTD. Despite being strongly correlated at a regional level, the two PET tracers carry some unique information across these conditions, as illustrated by the improvement in distinguishing bvFTD from svPPA when multi-modal data were jointly available to the classifier.

The correlation between regional distributions of neuroinflammation and protein aggregation supports a close relationship between these processes in FTD, mirroring recent evidence from Alzheimer’s disease that neuroinflammation is correlated with tau aggregation (Dani *et al.*, 2018), and extending this to TDP-43 associated diseases. Further, we confirm that this relationship is present throughout the disease course, by demonstrating it both *in vivo* with PET and post-mortem with immunohistochemistry. The partial correlations confirm that this association is over-and-above the effect of atrophy in FTD. While there were moderate associations between ligand binding and atrophy in some groups, in all cases these associations explained less of the variance than the association between ^11^C-PK-11195 and ^18^F-AV-1451, suggesting that neuroinflammation and protein aggregation may be more tightly associated with each other than either process is with volume loss. Similarly, in the post-mortem sample the density of cell nuclei explained some of the variance in the density of microglia, but additional variance was explained by the density of pathology. One interpretation of regionally co-localized neuroinflammation and protein aggregation is that microglial activation is an early pathophysiological process, which promotes or accelerates abnormal protein misfolding and aggregation. Microglia play a key role in orchestrating the innate immune response of the brain. They can be activated by misfolded proteins, and mediate responses through inflammatory pathways, cytotoxicity and changes in plasticity (Nakajima and Kohsaka, 2001; Nayak *et al.*, 2014). In neurodegenerative diseases, this state of activation may become chronic, dysfunctional, and toxic, contributing to pathogenicity (Pasqualetti *et al.*, 2015; Serrano-Pozo *et al.*, 2016).

There is corollary evidence for inflammation in FTD (Heneka *et al.*, 2014), from genetic (Guerreiro *et al.*, 2013; Rayaprolu *et al.*, 2013; Broce *et al.*, 2018), CSF (Sjogren *et al.*, 2004; Woollacott *et al.*, 2018), epidemiology (Miller *et al.*, 2013, 2016), post-mortem (Venneti *et al.*, 2008; Lant *et al.*, 2014) and animal studies (Yoshiyama *et al.*, 2007; Bhaskar *et al.*, 2010; Yin *et al.*, 2010). It is well established that an innate immune response, characterized by activated microglia, is a feature of the neuropathology of FTD (Lant *et al.*, 2014). Furthermore, mutations leading to haplo-insufficiency of GRN, a growth factor that has peripheral immune and central microglial regulatory functions (Petkau *et al.*, 2010; Yin *et al.*, 2010; Pickford *et al.*, 2011), produce FTD syndromes characterized by TDP-43 pathology. Expansions in C9ORF72 have effects on microglial function as well as neurons (O’Rouke *et al.*, 2016), and risk variants for FTD in TREM2 are associated with microglial activation (Giraldo *et al.*, 2013). Neuroinflammation is an early feature of pathophysiology in mouse models of tauopathy, where inflammatory changes precede the accumulation of aggregated tau (Yoshiyama *et al.*, 2007) and pro-inflammatory molecules increase tau hyperphosphorylation and aggregation (Bhaskar *et al.*, 2010). *In vivo* PET studies in small samples have shown that neuroinflammation anticipates atrophy in clinically established FTD (Cagnin *et al.*, 2004) and precedes both symptoms and the detectability of tau aggregation by PET in MAPT mutation carriers (Miyoshi *et al.*, 2010; Bevan-Jones *et al.*, 2019). Although neuroinflammation appears early in the pathogenesis of FTD and other neurodegenerative disorders, it remains unclear whether it is an independently initiating factor or whether it is induced by oligomeric proteins or pre-tangles.

Much of the evidence supporting the presence of inflammation in FTD comes from *ex vivo* studies, and indeed we provide further post-mortem evidence here. The need to improve our understanding of this process during life has led to the development of PET radioligands, but there is some controversy over the optimum ligand for imaging activated microglia. PET ligands, which target TSPO, have long been the mainstay of imaging microglia. However TSPO expression patterns in microglia are complex and the functional effects of different microglial phenotypes are incompletely understood (Gomez-Nicola and Perry, 2015). TSPO is also expressed by other cell types, notably astrocytes (McCarthy and Harden, 1981). However, in favour of the use of ^11^C-PK-11195 is its demonstrated selectivity for activated microglia over quiescent microglia and reactive astrocytes (Banati, 2002), its relative insensitivity to common polymorphisms in TSPO compared to second generation TSPO ligands (Zhang, 2015; Stefaniak and O’Brien, 2016), and the fact that it has well established methods of non-invasive kinetic analysis (Turkheimer *et al.*, 2007; Passamonti *et al.*, 2018). ^11^C-PK-11195 has been used in studies of other neurodegenerative diseases (Edison *et al.*, 2013; Varley *et al.*, 2015; Stefaniak and O’Brien, 2016; Passamonti *et al.*, 2018). There remain some disadvantages, including non-specific binding and low brain penetration. Whilst its limited signal-to-noise ratio might explain previous negative studies using ^11^C-PK-11195, this does not undermine positive findings such as those shown here, especially within our multivariate analyses that explicitly control for differences in ligand penetration and affinity. When interpreting the meaning of increased ^11^C-PK-11195 binding one must consider the potential contribution from reactive astrocytes expressing upregulated TSPO, and while increased ^11^C-PK-11195 binding may still be interpreted as immune activation, a causal role of inflammation in human dementia is yet to be proven by interventional studies.

In contrast to ^11^C-PK-11195, the ^18^F-AV-1451 binding provides an ambiguous signal despite protein aggregation being an essential feature of FTD and many other dementias. We propose that ^18^F-AV-1451 binding is a proxy measure of aggregated non-amyloid-β proteins in FTD. In Alzheimer’s disease the sensitivity of *in vivo* imaging with ^18^F-AV-1451, and its affinity for tau in neurofibrillary tangles, is well established and has contributed significantly to our understanding of its pathogenesis and progression. However, the situation in FTD is more complex due in part to pathological heterogeneity and the differential affinity for tau aggregates arising from different isoforms and with different ultrastructure (Jones *et al.*, 2018). The molecular target in FTD associated with TDP43 is also as yet undetermined, but is unlikely to be TDP43 itself (Marquié *et al.*, 2015; Lowe *et al.*, 2016; Sander *et al.*, 2016). This heterogeneity is problematic for univariate regional analyses, and although six of our patients have genetic mutations, and six others were amyloid biomarker negative (Tables 1 and 2), we cannot definitively state the majority of patients’ pathological type ante-mortem.

The molecular targets for ^18^F-AV-1451 binding remain controversial, and several off-target binding possibilities need to be considered. Supporting our use of ^18^F-AV-1451 as a marker of non-amyloid-β protein aggregation, previous post-mortem work has demonstrated some binding to FTLD pathologies, albeit at a lower magnitude than that seen with Alzheimer’s pathology (Marquié *et al.*, 2015; Lowe *et al.*, 2016; Mcmillan *et al.*, 2016; Sander *et al.*, 2016). This is corroborated by *in vivo* studies of patients with a straight filament 4-repeat tauopathy and clinical FTD resulting from *MAPT* mutations, showing binding in areas typically affected in FTD and affected at post-mortem (Bevan-Jones *et al.*, 2016; Smith *et al.*, 2016), and by the elevated binding in the affected brain regions of patients with svPPA (Bevan-Jones *et al.*, 2018*a*, Makaretz *et al.*, 2018) and bvFTD due to *C9orf72* expansions (Bevan-Jones *et al.*, 2018*b*), who have TDP-43 rather than tau pathology. However, even within genetically determined FTD, binding affinity varies according to different tau isoforms and strains (Jones *et al.*, 2018) supporting varying affinity to different morphologies of tau.

^18^F-AV-1451 can have other non-tau, non-TDP-43, targets such as neuromelanin (Marquié *et al.*, 2015) and mono-amine oxidase (MAO) (Vermeiren *et al.*, 2018), and such off-target binding is an important caveat in clinical studies using ^18^F-AV-1451 (Baker *et al.*, 2019). Neuromelanin in the catecholaminergic brainstem nuclei is a particular concern in studies of brainstem tau pathology in progressive supranuclear palsy, and artefactual binding in Parkinson’s disease. However, neuromelanin is not expressed by cortex even in tauopathies (Passamonti *et al.*, 2018) and is unlikely to account for our FTD results. MAO subtypes are expressed by both neurons (MAOA, especially in basal ganglia) and reactive astrocytes (mainly MAOB) (Fowler *et al.*, 2005; Ben Haim *et al.*, 2015). If ^18^F-AV-1451 binding were driven by ‘off target’ binding to reactive astrocytes, which are induced by activated microglia (Liddelow *et al.*, 2017), this would provide further evidence for the importance of neuroinflammation in FTD, but it would undermine the inference we make on the relationship between protein aggregation and inflammation. Although ^18^F-AV-1451 has weak affinity for both types of MAO (Drake *et al.*, 2019), selective blockade of MAOA and MAOB leads to only minor changes in the estimated cortical binding from PET in non-human primates (Drake *et al.*, 2019), while clinical treatment with MAO inhibitors does not significantly change estimated ^18^F-AV-1451 uptake in humans (Hansen *et al.*, 2018; Smith *et al.*, 2018). Moreover, the presymptomatic dissociation of ^11^C-PK-11195 and ^18^F-AV-1451 binding (Bevan-Jones *et al.*, 2019) argues strongly against simple cross-affinity. Similarly, in our own post-mortem cases we see regional co-localization of both FTD-tau and FTD-TDP-43 aggregates with microglial activation, to at least as great a degree as is observed in Alzheimer’s disease (Fig. 6). Together, these observations suggest that neuroinflammation and protein aggregation co-occur in symptomatic stages, but in early stage disease neuroinflammation can occur in the absence of ^18^F-AV-1451 binding. Neuroinflammation and protein aggregation are at least partially dissociable processes by current PET ligands.

In the face of uncertainty about molecular targets and variations in affinity, it is important to emphasize that through our classification analysis we focus on distribution rather than quantification of binding, using a non-parametric method that is insensitive to absolute binding values and instead reflects the spatial pattern of binding. This takes into account the potential differences in affinity of ^18^F-AV-1451 for different protein targets. Overall, whilst it is clear that ^18^F-AV-1451 does not bind exclusively to tau aggregates, the distribution of binding regionally co-localizes and varies with that expected of aggregated protein in these diseases, and post-mortem immunohistochemistry of tau. Indeed, ^18^F-AV-1451 may provide a usefully non-selective marker of non-amyloid-β aggregated protein, whether tau or TDP-43, allowing *in vivo* examination across the spectrum of sporadic FTD syndromes. Whilst in the complex setting of FTLD we interpret ^18^F-AV-1451 binding as a non-specific marker of non-amyloid-β neuropathology, the biological relevance of elevated binding in non-Alzheimer’s disease neurodegenerative disease remains incompletely understood. Further work examining ^18^F-AV-1451 binding across large post-mortem cohorts of FTLD pathology will be required to independently validate our hypothesis.

The main limitation of this study is group size which, although larger than most previous PET studies in FTD, is still small for each individual diagnosis. The small sample size reduces the power of the study to find parametric group differences in binding, particularly given that both ligands have a degree of insensitivity to their targets, as well as limiting the ability to detect associations with clinical features and severity. Characterization of the groups is also limited in that the genotyping and amyloid assays were based on clinical indications and consent: we did not directly examine amyloid status in all individuals and whilst there is a mix of both genetic and sporadic cases, we did not genotype every participant. The inability to perform pathological subtyping *in vivo* makes interpretation of results more difficult in view of the generally poor relationship between phenotype and underlying neuropathology in FTD. Consequently, we cannot use the clinical diagnostic groups alone to draw conclusions about the relationship between microglial activation and specific forms of protein aggregation. We are also limited in the inferences about the predilection for immune dysregulation in a particular neuropathological subtype, such as the relationship suggested between immune dysfunction and FTLD-TDP-43 (Miller *et al.*, 2013, 2016), except for the cases with genetic mutations.

To conclude, we provide *in vivo* evidence for neuroinflammation in FTD, which has a close relationship with ^18^F-AV-1451 binding, taken in this study to represent a marker of either FTLD-tau or FTLD-TDP-43 neuropathology. PET measurement of inflammation provided a more accurate classification of syndromes than did protein aggregation emphasizing its potential importance in shaping the clinical and neuropathological patterns of the diverse clinical syndromes of FTD. A causal role for neuroinflammation in neurodegeneration would inform future drug targets and potential clinical trials in FTD. Our findings therefore warrant further longitudinal mechanistic investigation into the role of neuroinflammation in early-stage neurodegeneration, its relationship to specific protein aggregation and to clinical progression.

## Supplementary Material

awaa033_Supplementary_Data

## Abbreviations

BP_ND_ =: non-displaceable binding potential;
bvFTD =: behavioural variant FTD;
FTD =: frontotemporal dementia;
nfvPPA =: non-fluent variant primary progressive aphasia;
svPPA =: semantic variant primary progressive aphasia
