# Supplementary material for: Neuroinflammation and protein aggregation co-localize across the frontotemporal dementia spectrum
Source: Brain. 2020 Mar 17;143(3):1010–26. doi: 10.1093/brain/awaa033 (PMC7089669; doi:10.1093/brain/awaa033)
Supplement: awaa033_Supplementary_Data [file awaa033_supplementary_data.zip › awaa033-suppl_data/awaa033_Supplementary Fig. 1.pdf]

Supplementary material

Supplementary figure 1: Scatter plot of the raw regional mean BP_{ND} for [^{11}C]PK-11195 against regional mean BP_{ND} of [^{18}F]AV-1451 between the control groups.

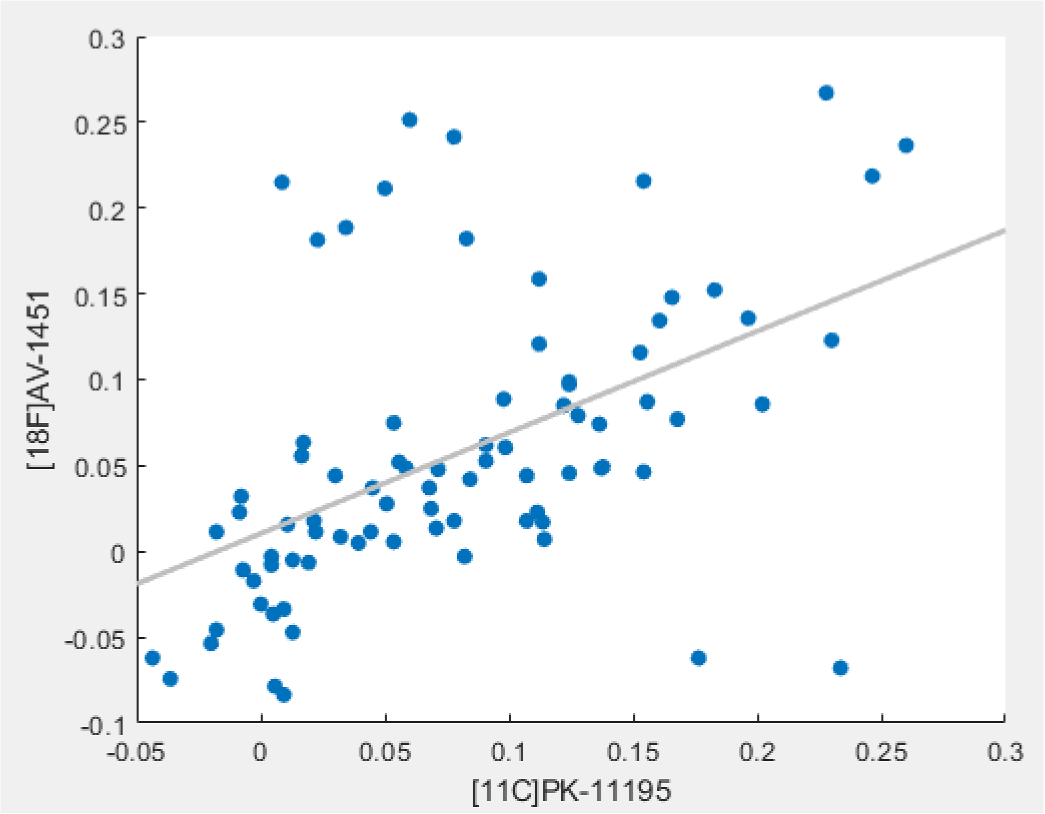

Supplementary figure 2: Scatter plot of the regional mean BP_{ND} for [11C]PK-11195 against regional mean BP_{ND} of [18F]AV-1451 for all disease groups, split by brain region. Patients with svPPA are represented in torquoise, those with nfvPPA in purple, and those with bvFTD in yellow. All values are corrected for control binding. Trend lines are emboldened where an ANCOVA demonstrated a significant association between the binding of the ligands, controlling for diagnostic group.

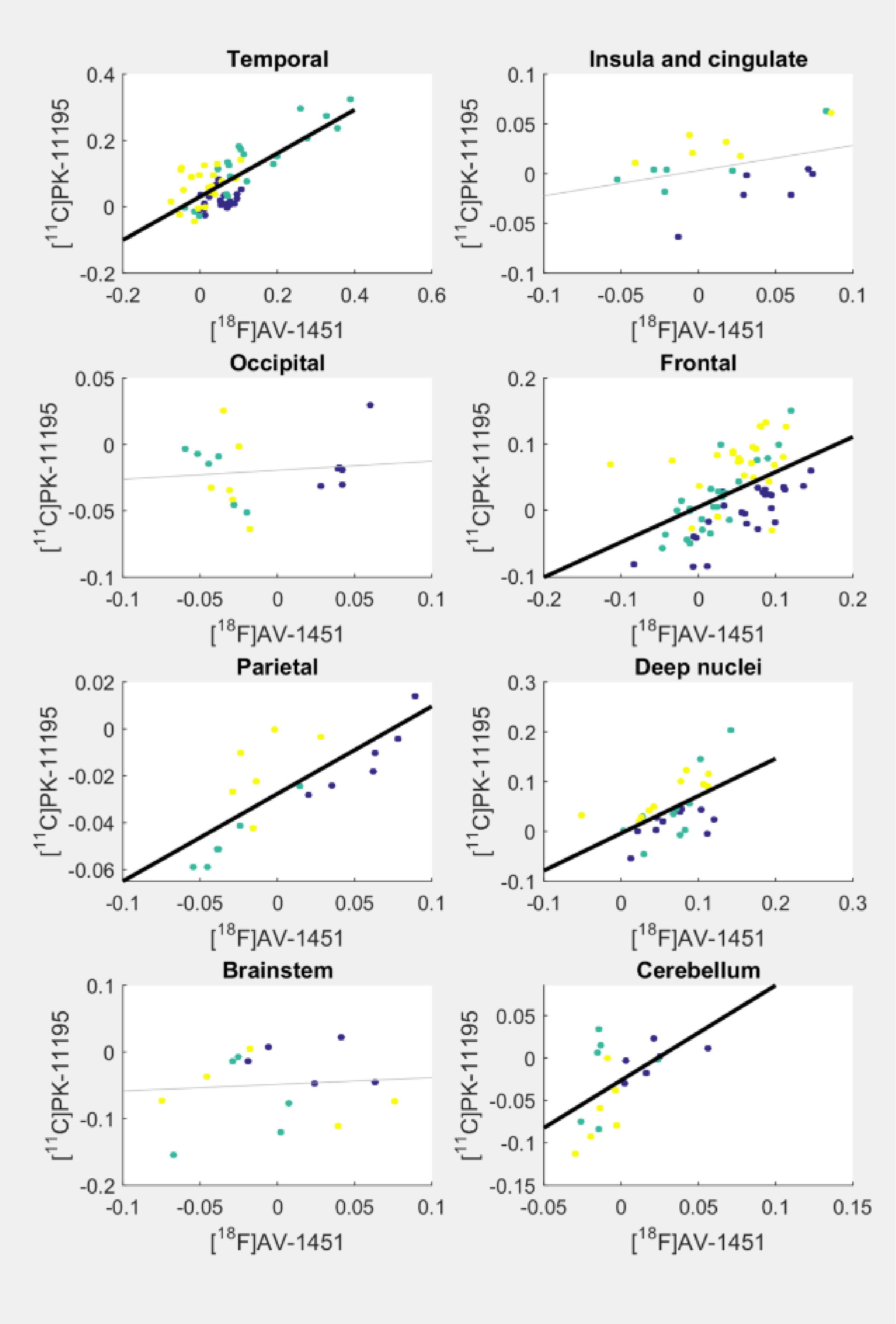

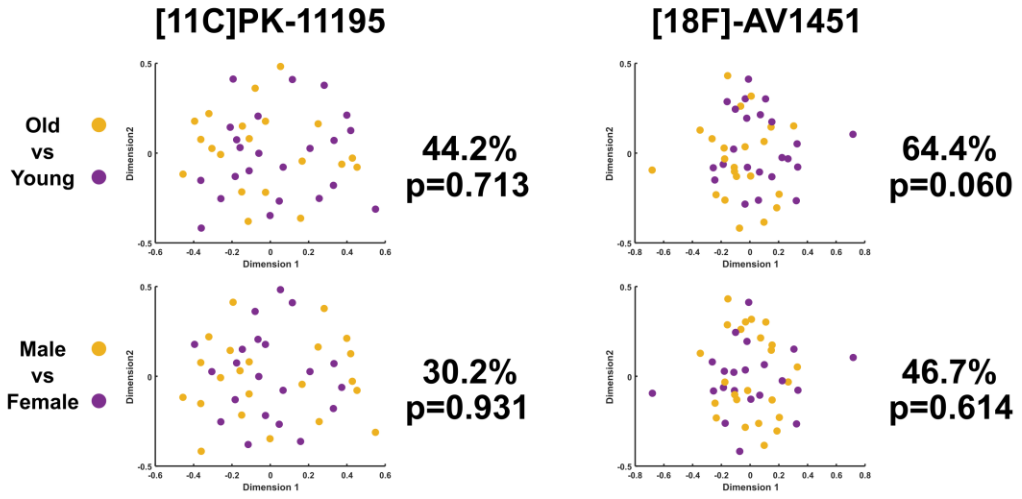

Supplementary figure 3: Pairwise classification accuracy for each ligand ([^{11}C]PK-11195 on the left, [^{18}F]AV-1451 on the right) for age and sex. Age classification was against a binary split in which 'Old' was defined as greater than the median age (67 for both ligands) and 'Young' was defined as less than or equal to this age. The method was unable to classify either factor better than chance, suggesting that these factors did not add any significant bias to the analysis presented in figure 4.

Diagnosis	Brain Weight	Age (yrs)	Sex
FTLD-TDP (C)	880	77	F
FTLD-TDP (C)	750	77	F
FTLD-TDP (C)	1202	90	M
FTLD-TDP (A)	1096	68	F
FTLD-TDP (A)	1300	66	M
FTLD-TDP (A)	920	70	M
FTLD-P (Pick's disease tau)	870	71	M
FTLD-P (Pick's disease tau)	973	72	M
FTLD-P (Pick's disease tau)	928	70	F
AD	1238	63	M
AD	1027	96	F
AD	1155	89	M

Supplementary table 1: Brain weight, age and sex for the 12 *post mortem* cases.
