# Supplementary figures and images for: Neuroinflammation and protein aggregation co-localize across the frontotemporal dementia spectrum
Source: Brain. 2020 Mar 17;143(3):1010–26. doi: 10.1093/brain/awaa033 (PMC7089669; doi:10.1093/brain/awaa033)

**Temporal**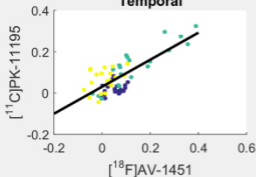**Insula and cingulate**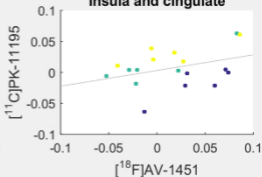**Occipital**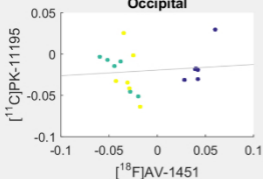**Frontal**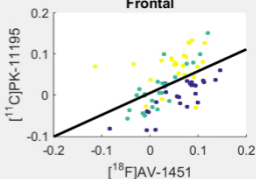**Parietal**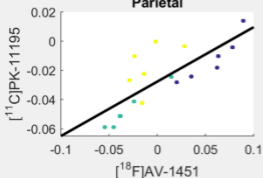**Deep nuclei**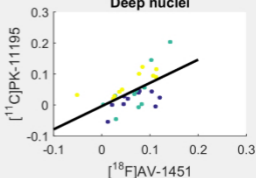**Brainstem**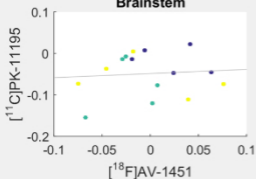**Cerebellum**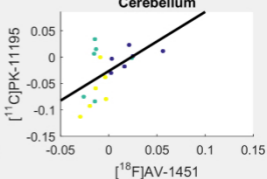

Supplement: awaa033_Supplementary_Data [file awaa033_supplementary_data.zip › awaa033-suppl_data/awaa033_Supplementary Fig. 2.pdf]

[11C]PK-11195

Old  
vs  
Young

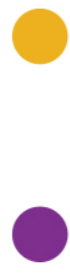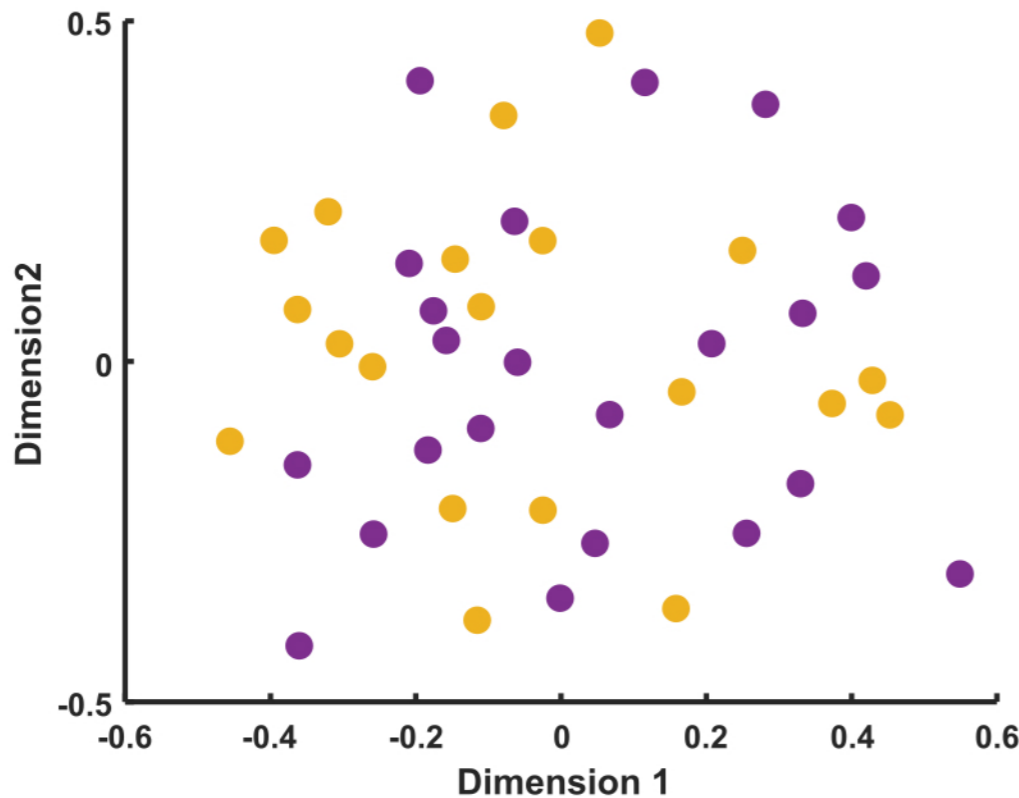

44.2%  
p=0.713

Male  
vs  
Female

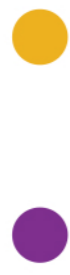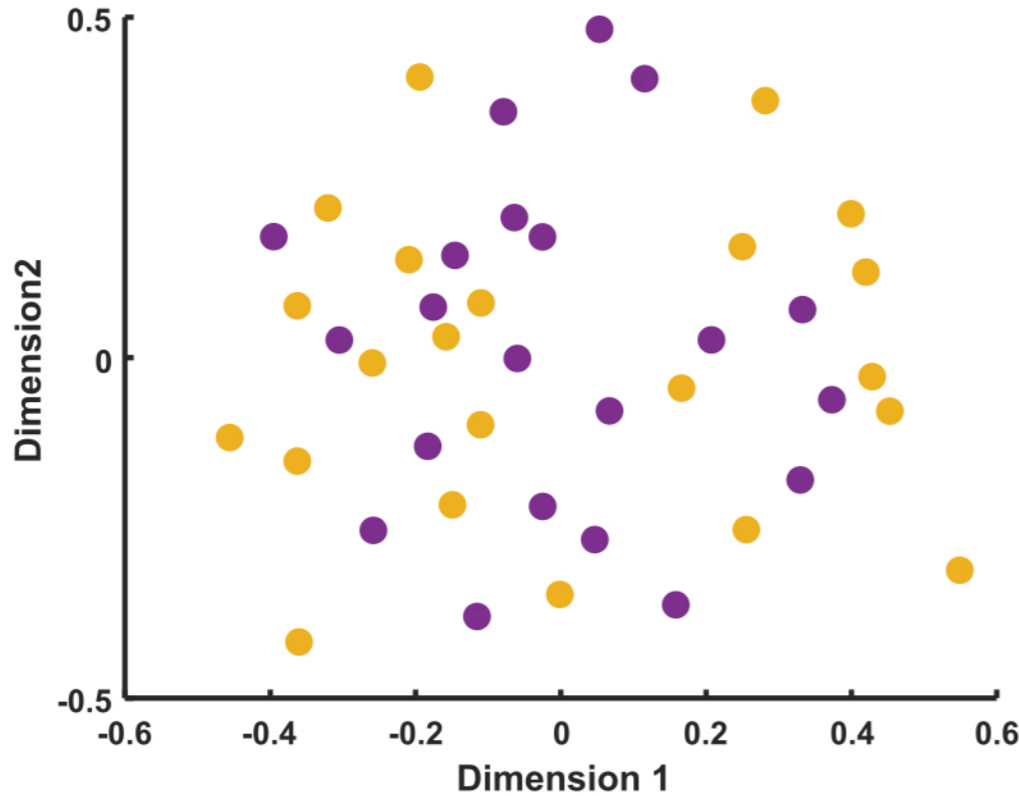

30.2%  
p=0.931

[18F]-AV1451

64.4%  
p=0.060

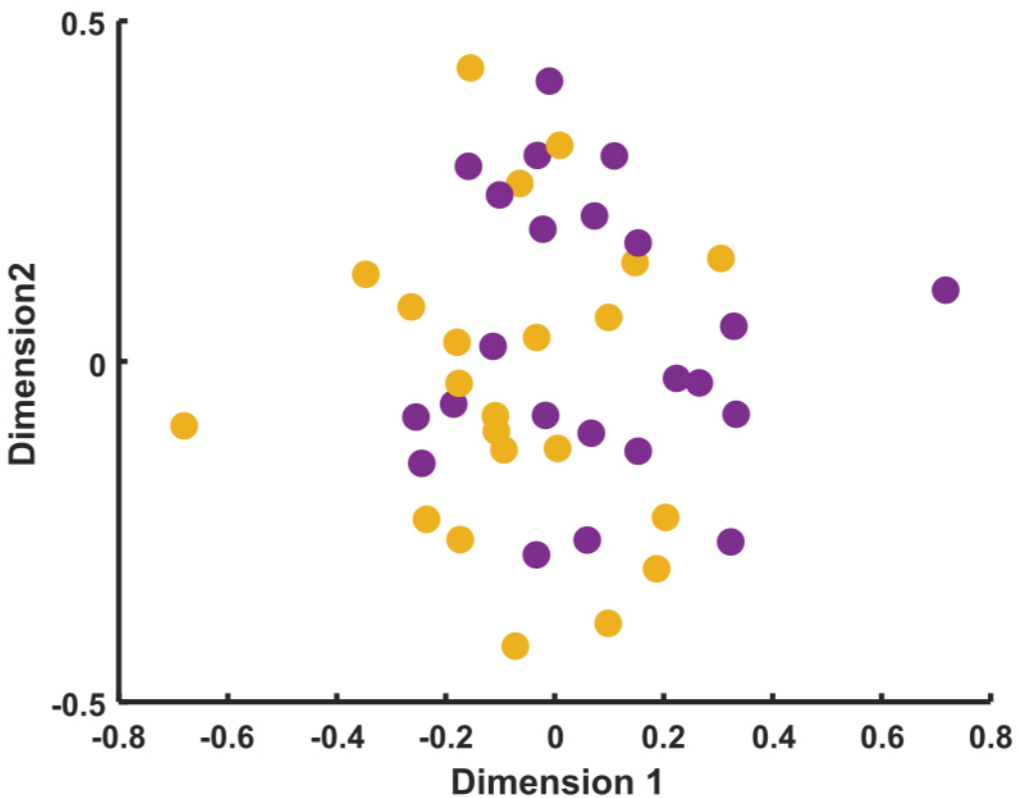

46.7%  
p=0.614

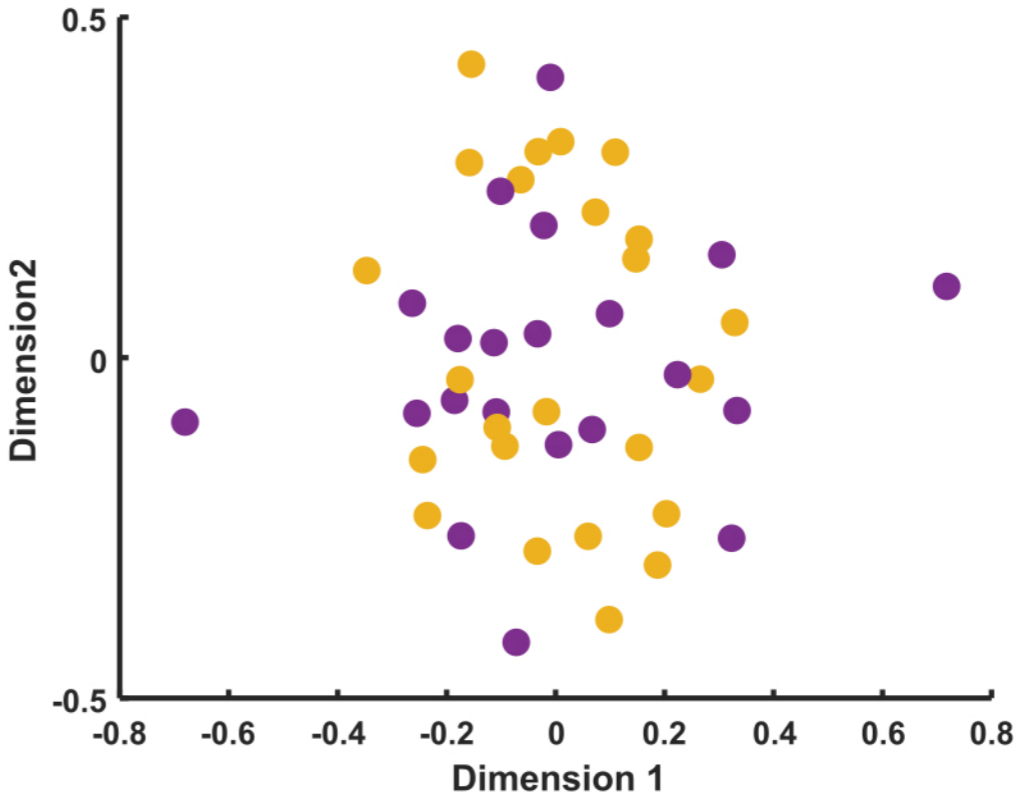

Supplement: awaa033_Supplementary_Data [file awaa033_supplementary_data.zip › awaa033-suppl_data/awaa033_Supplementary Fig. 3.pdf]
